# Quantification of the antibody response to *Propionibacterium acnes* in a patient with prosthetic valve endocarditis: – a case report

**DOI:** 10.1186/s12879-016-1522-2

**Published:** 2016-04-29

**Authors:** T. Herren, M. A. Middendorp, R. Zbinden

**Affiliations:** Department of Medicine, Limmattal Hospital, Urdorferstrasse 100, CH-8952 Schlieren, Switzerland; Department of Surgery, Kantonsspital, Im Ergel 1, CH-5404 Baden, Switzerland; Microbiological Laboratory, Limmattal Hospital, Urdorferstrasse 100, CH-8952 Schlieren, Switzerland

**Keywords:** Infective endocarditis, Prosthetic heart valve, *Propionibacterium acnes*, Serologic tests, Bacteremia, Contamination

## Abstract

**Background:**

The isolation of *Propionibacterium acnes* in blood cultures is often considered a contaminant. On rare occasions, *P. acnes* can cause severe infections, including endocarditis and intravascular prosthesis-associated infections. To evaluate the discrimination between a contaminant and a clinically relevant infection we used an Ouchterlony test system to quantify the antibody response to *P. acnes* in a patient with a proven *P. acnes* endocarditis.

**Case presentation:**

We report on a 64-year-old Caucasian man who developed *P. acnes* endocarditis four years following a composite valve-graft conduit replacement of the aortic root. Bacterial growth in blood cultures was detected after an incubation period of 6 days. However, the antibody titer to *P. acnes* was 1:8 at the time of diagnosis and declined slowly thereafter over 2½ years. The patient’s response to the antibiotic treatment was excellent, and no surgical re-intervention was necessary.

**Conclusion:**

The working hypothesis of infective endocarditis can be substantiated by serologic testing, which, if positive, provides one additional minor criterion. Moreover, quantification of the antibody response to *P. acnes*, though not specific, may assist in the differentiation between contaminants and an infection. This quantification may have implications for the patient management, e.g. indication for and choice of the antibiotic therapy.

## Background

*Propionibacterium acnes* is often considered a contaminant when it grows in blood culture, akin to the growth of coagulase-negative staphylococci, *Bacillus* sp., and *Corynebacterium* sp. In a series of 522 patients with *P. acnes* bacteremia, only 18 (3.5 %) were considered to have a clinically significant bacteremia [1]. Diagnosis of the latter required at least two sets of blood cultures showing growth of *P. acnes*, and the patients had to have a systemic inflammatory response syndrome [1]. In equivocal cases, the availability of a serologic test for *P. acnes* would assist in the differentiation between true infection and contaminant bacterial growth. Moreover, the slow growth of *P. acnes* in blood cultures requires prolonged incubation periods of up to two weeks [2]. In patients with suspected infective endocarditis, antibiotic therapy often cannot be withheld for such a long duration. A higher diagnostic precision is desired in such circumstances, and a positive serologic test for *P. acnes* would confirm the diagnosis. Furthermore, the concentration time course of the antibody titers can show a decline in case of the elimination of *P. acnes,* or a late rise indicating a relapse. Antibodies against *P. acnes* were characterized using enzyme-linked immunosorbent assays (ELISAs) in patients with acne vulgaris [3] and in patients with prostatitis and benign prostatic hyperplasia [4]. To our knowledge, serologic testing in patients with suspected *P. acnes* endocarditis has not been reported before. Thus, we describe a concentration time graph of the antibody titer to *P. acnes* in a patient with proven infective endocarditis. The potential clinical application of serologic testing is discussed, and specific features of *P. acnes* endocarditis, especially regarding its sometimes difficult diagnosis, are outlined.

## Case presentation

A 64-year-old Caucasian man had severe regurgitation of the tricuspid aortic valve due to an aneurysm of the ascending aorta, which involved the sinus of Valsalva. Composite valve-graft conduit replacement of the aortic root was performed 4 years ago. The patient suffered a transient ischemic attack with a thrombotic occlusion of a branch of the left middle cerebral artery 18 months postoperatively, most likely due to subtherapeutic oral anticoagulation (international normalized ratio [INR] 1.8). Transesophageal echocardiography (TEE) did not show vegetations on the mechanical aortic valve and the concentration of C-reactive protein was 1 mg/L (normal value <5). Aspirin was then added to the vitamin K antagonist phenprocoumon, and the patient successfully self-monitored his INR values with a target range of 2.0–3.0. He did not use skin disinfectants, and he had no acne.

**Fig. 1:**
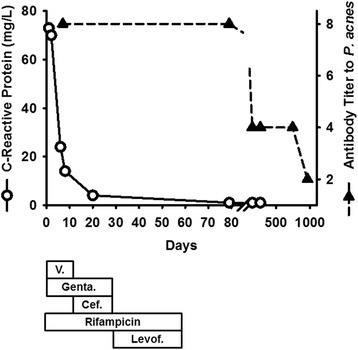
Concentration time graph of the C-reactive protein (open circles, solid line) and the anti - *P. acnes* antibody titer (filled triangles, dashed line). The latter was measured using the Ouchterlony immuno-diffusion test. The antibiotic therapy is shown in the horizontal bars below the graph (V. = Vancomycin, Genta. = Gentamicin, Cef. = Ceftriaxone, Levof. = Levofloxacin)

**Fig. 2:**
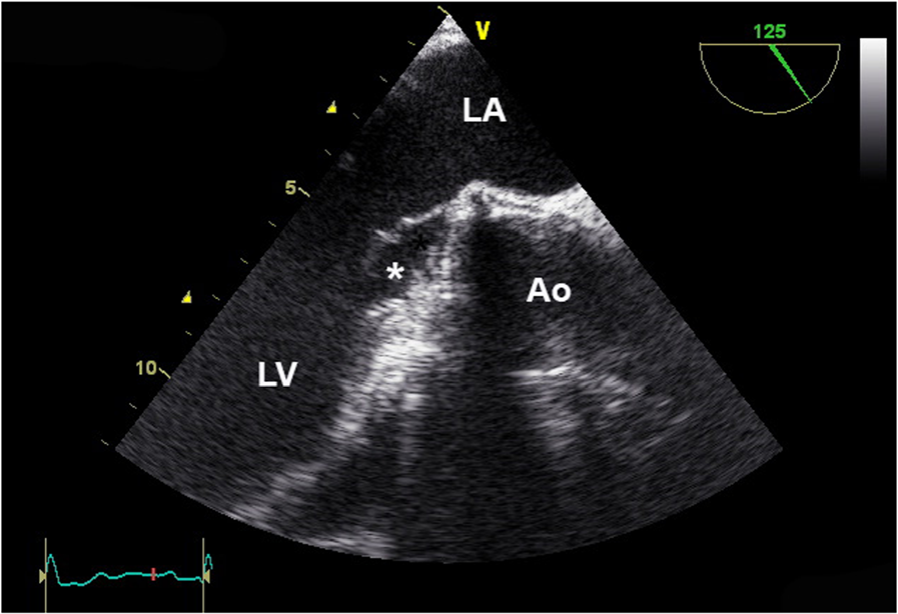
Photograph obtained during transesophageal echocardiography (long axis view of the left ventricular outflow tract). Two vegetations (*) are visible on the mechanical bileaflet aortic valve, protruding into the left ventricular outflow tract in diastole. Ao = aorta, LA = left atrium, LV = left ventricle

A wasp sting to his upper lip was managed conservatively one month prior to hospitalization, and subdued sounds of his mechanical aortic valve were noticed by the patient. No dental procedures were performed in the six months prior to the sting. He became febrile up to 38 °C, had night sweats and felt ill. Upon admission, the patient was in good general condition. His body temperature was 37.3 °C; his blood pressure was 137/94 mmHg; and his pulse rate was 79 bpm. There was a 1/6 systolic murmur and a metallic hue to the second heart sound. There were no peripheral emboli in the skin. The spleen was not enlarged. Laboratory results showed an elevated concentration of C-reactive protein (73 mg/L, Fig. 1) and a normal leukocyte count (7.3×10^9^/L [normal values 4.0–9.8]). The kidney function (creatinine 90 μmol/L [62–110]) and the liver function tests (aspartate transaminase [AST] 29 U/L [<38]) were normal. The INR value was 2.4, consistent with therapeutic anticoagulation with phenprocoumon. The lactic dehydrogenase was slightly elevated (655 U/L [<248]). An infective endocarditis was suspected clinically, and TEE was performed. Vegetations on the mechanical bileaflet aortic valve were seen (Fig. 2), but there was no perivalvular abscess formation. Ten blood cultures (Bactec^TM^ 9050, Becton Dickinson, Franklin Lakes, NJ) were inoculated with 10 ml blood samples, five cultured under aerobic and five under anaerobic conditions. All blood cultures were obtained before starting the antibiotic therapy. Initially, no bacterial growth was observed. Broad range polymerase chain reaction (PCR) using 16S rDNA primers failed to identify bacteria in blood samples. One anaerobic blood-culture showed growth of a Gram-positive rod after 6 days, which was sent to the reference laboratory for further testing. The identification of *P. acnes* was performed according to standard procedures with conventional tests. The Gram-positive rods were catalase positive, showed a positive CAMP reaction, reduced nitrate and produced indole, but were lipase and lecithinase negative. Utilizing gas-liquid chromatography, the production of propionic acid from the glucose broth was detected. In four of 5 anaerobic blood cultures bacteria grew within 12 days, and biochemical analysis again identified *P. acnes*; all aerobic blood cultures remained sterile. Furthermore, the antibody response to this bacterium was quantified (Fig. 1). According to the modified Duke criteria, a definitive diagnosis of infective endocarditis was confirmed [5]. The initial antibiotic treatment included vancomycin and gentamicin i.v. plus rifampicin p.o. After the identification of the *P. acnes,* vancomycin was stopped and ceftriaxone was started. The intravenous therapy was stopped after four weeks, and levofloxacine plus rifampicin were prescribed for an additional four weeks of oral antibiotic therapy. No bacteria grew in the blood cultures following the completion of the antibiotic therapy. A follow-up TEE showed no vegetations on the mechanical aortic valve, and the patient continues to be well. He consistently uses skin disinfectants prior to obtaining a blood specimen for his INR measurements.

Sera drawn at different time points were tested for antibodies against *P. acnes* by the Ouchterlony assay [6] in the following 2½ years. For the antigen preparation of the *P. acnes* strain (ATCC 6916, Manassas, VA) colonies of 20 sheep blood agar plates were suspended in a tube with 10 ml of phosphate buffered saline (pH 7.2) and sonicated in a cup horn sonifier (60 W, Branson Sonic Power Co., Danbury, CT) 8 times for one minute. The tube was centrifuged at 2000x*g* for 10 min following overnight storage at 4 °C. Aliquots of the supernatant, each containing100 μL, were stored at −80 °C. The patient’s sera were tested against this antigen with the Ouchterlony test [7]. The highest dilution of the serum that still precipitated the *P. acnes* antigen in the Ouchterlony test system (ID Plates Cleargel, IMMY, Norman, OK) represented the titer of this serum. The titers decreased during the following 2½ years indicating the clearing of the bacteria without relapse (Fig. 1).

## Discussion

*Propionibacterium* spp. are Gram-positive, slow-growing anaerobic, non-spore-forming rods. They are part of the normal flora of the skin, nasopharynx, oral cavity, and genitourinary tract. Generally thought to be nonpathogenic in humans, they can cause infective endocarditis, especially in carriers of mechanical heart valves and pacemakers or implantable cardioverter defibrillators (ICDs) [8]. This may be due to their ability to adhere to foreign body surfaces and produce a biofilm. In a series of 58 patients with infective endocarditis due to *Propionibacterium* spp. published in 2009, prosthetic valve endocarditis occurred in 67 % of the patients and was the most common presentation of *Propionibacterium* spp. infection. In addition to antimicrobial treatment, 81 % of the patient underwent cardiac surgery (due to the propensity of the organism to form an abscess [9]), and the overall mortality rate was 16 %[8]. The patient described above was cured with antibiotic therapy alone. Most of the infections of mechanical heart valves occur at the time of surgery. With high inocula, a short incubation period is expected. However, *P. acnes* may reside intracellularly in macrophages and remain there dormant for years [10]. In our patient, the infection may also have occurred during the frequent skin punctures necessary for the determination of the level of anticoagulation [11]. *Propionibacterium* endocarditis may be difficult to diagnose. The median incubation period required for blood cultures to become positive is 7 days (range 5–14 days) [2]. Therefore, a prolonged incubation period of two weeks is proposed in cases of suspected infection with *Propionibacterium* spp. Additional diagnostic methods are often necessary. PCR to detect 16S rDNA in blood is used in cases of blood culture-negative infective endocarditis. However, its sensitivity is lower than that of blood culture methods, and blood specimens need to be pretreated to remove background bacterial DNA contamination [12]. Gas-liquid chromatographic examination of subcultured bacterial colonies may assist in the detection of anaerobic bacteria [13]. In blood culture-negative infective endocarditis, which occurs in 2.5–31 % of all cases of infective endocarditis, serologic techniques may be useful [14], and are established to diagnose infections including *Brucella* spp., *Coxiella burnetii* [5], *Bartonella* spp., *Mycoplasma* spp., and *Legionella* spp.[15]. In the patient described above, an antibody response to *P. acnes* was quantified (Fig. 1), showing a titer of 1:8 at the time of diagnosis and a gradual decline thereafter. Therefore, this antibody could potentially facilitate the early diagnosis of infective endocarditis due to *P. acnes*. When uncertainty exists regarding whether the growth of *P. acnes* in blood cultures is a contaminant, as occurs in > 95 % of cases [1], or whether it has a true pathogenetic role, a detectable antibody titer would support the latter. However, increased antibody titers against *P. acnes* have been reported in diseases such as acne vulgaris [16] or benign prostatic hyperplasia [4], lowering their specificity. In patients with acne a time course of the serology can detect a rising antibody titer suggesting *P. acnes* infection.

Treatment of *P. acnes* infective endocarditis primarily consists of a β-lactam antibiotic, often combined with an aminoglycoside [8], even though P. acnes is frequently resistant to the latter [17]. Oral rifampicin must be added because of its ability to penetrate the bacterial biofilm [8] and may be combined with a chinolone antibiotic [18]. In vitro data suggest that the combination of daptomycin with rifampicin is highly active against *P. acnes* biofilms [19]. Long-term antibiotic therapy is required for the successful treatment of infectious endocarditis due to *P. acnes*. The treatment effect is mirrored by the decreasing antibody titer.

## Conclusions

1. In patients with suspected *P. acnes* infective endocarditis, a high antibody titer to the bacterium is useful diagnostic adjunct. According to the modified Duke criteria, serological evidence of active infection represents a minor criterion for the diagnosis of infective endocarditis [5].
2. Serologic testing may be useful in cases of *P. acnes* bacteremia to differentiate between blood culture contamination and a clinically relevant infection.
3. The Ouchterlony test used in the present case report allows the analysis of the antibody titer to *P. acnes* over time. Therefore, it may be useful even in acne patients, even though the specificity will be lower. Validation in a larger clinical cohort including blood donors and patients with acne as controls is important.

### Ethics and consent to publish

The patient was identified during routine clinical practice and consented to give venous blood samples after elaborate information. Involvement of the ethical committee of the Canton of Zurich was not considered necessary, since the project was not based on a study protocol, and is not classified as research by the Swiss Federal Act on Research on Human Beings. Written informed consent was obtained from the patient for publication of this case report and all accompanying images. A copy of the written consent is available for review by the editor of this journal.

### Availability of supporting data

The data set supporting the results of this article is depicted in Fig. 1. *P. acnes* used for the Ouchterlony test system was from the American Type Culture Collection (ATCC 6916), headquartered in Manassas, VA.

## Abbreviations

AST: aspartate transaminase
CAMP: Christie Atkins Munch-Petersen
ELISA: enzyme-linked immunosorbent assay
ICD: internal cardioverter defibrillator
INR: international normalized ratio
PCR: polymerase chain reaction
rDNA: ribosomal deoxyribonucleic acid
TEE: transesophageal echocardiography
